# Three‐Year Visual Quality Evaluation in Children Using Orthokeratology Lenses: A Retrospective Study With OPD‐Scan III Measurements

**DOI:** 10.1155/joph/6682142

**Published:** 2026-05-31

**Authors:** Shang Li, Yihan Guo, Lei Tian, Qianru Wu, Ao Li, Ying Jie

**Affiliations:** ^1^ Beijing Institute of Ophthalmology, Beijing Tongren Eye Center, Beijing Tongren Hospital, Capital Medical University, Beijing Ophthalmology and Visual Sciences Key Laboratory, Beijing, 100730, China, trhos.com

**Keywords:** myopia, OPD-Scan III measurements, orthokeratology, visual quality

## Abstract

**Purpose:**

To evaluate long‐term visual quality outcomes, refractive changes, and safety during orthokeratology lens wear in children with myopia over a 3‐year period.

**Methods:**

We conducted a retrospective analysis of 130 myopic children aged 8–18 years who wore corneal refractive therapy (CRT) lenses from February 2021 to November 2024. Participants underwent comprehensive ocular examinations, including assessments of visual acuity, refractive error, axial length, corneal thickness, and visual quality parameters at baseline, 1.5 years, and 3 years. Data were analyzed with repeated measures ANOVA, and post hoc comparisons were conducted using the Bonferroni test.

**Results:**

Over the three‐year period, there was a slight increase in myopic refractive error and axial length (spherical equivalent: from −1.36 ± 1.17D to −1.69 ± 1.24D; cylindrical power: from −0.70 ± 0.83D to −1.18 ± 0.99D; axial length: from 24.62 ± 0.83 to 25.08 ± 0.85 mm). Overall ocular visual quality improved significantly, with increases in Strehl ratio and modulation transfer function (*p* < 0.001), while root mean square (RMS) of total ocular aberrations decreased (*p* < 0.001). After 1.5 years of wear, the corneal high‐order aberration (HOA) RMS, corneal spherical aberration (SA) RMS, corneal coma RMS, and corneal tilt RMS all significantly increased (*p* < 0.0167), while corneal HOAs in the 5‐mm optical zone were generally greater than those in the 3‐mm optical zone (*p* < 0.05). Total intraocular aberration RMS decreased significantly in both zones (*p* < 0.001). No significant changes were observed in corneal endothelial cell density, and no cases of microbial keratitis or other vision‐threatening complications occurred.

**Conclusion:**

Long‐term CRT lens wear was associated with improved overall and intraocular visual quality, despite increased corneal HOA, with better optical performance in the 3‐mm zone. The observed axial elongation and refractive changes over 3 years were broadly consistent with previously reported orthokeratology outcomes. CRT lenses also demonstrated a favorable safety profile in this cohort.

## 1. Introduction

Myopia is a global public health issue that affects approximately one‐third of children and adolescents, and it is projected that by 2050, the number of individuals with myopia will reach 740 million [1, 2]. Myopia not only affects daily activities and academic performance in children but can also lead to a series of serious ocular complications, such as retinal detachment and macular degeneration, posing a severe threat to vision health [3, 4].

Multiple myopia control strategies exist, encompassing environmental (e.g., increased outdoor time), pharmaceutical (e.g., low‐dose atropine), and optical interventions [5, 6]. Among optical treatments, orthokeratology has become particularly prevalent over the past decade, demonstrating myopia control efficacy of approximately 30% to 60% [7–10]. As a specialized form of orthokeratology, corneal refractive therapy (CRT) lenses are widely used in pediatric myopia management [11]. They work by reshaping the cornea, flattening the central corneal area to correct myopia [12, 13]. Compared with traditional spectacle lenses, CRT lenses not only provide effective refractive correction but also have been reported to slow myopia progression in children and adolescents in previous studies [14, 15].

As a type of orthokeratology lens, CRT lenses have distinct design and material characteristics that support their clinical use. They utilize an advanced reverse geometry design [16], allowing for more precise corneal reshaping and providing more stable visual outcomes. This design creates a central corneal treatment zone surrounded by a midperipheral steepening area, which contributes to refractive correction and peripheral myopic defocus. Moreover, CRT lenses are made from highly oxygen‐permeable materials with excellent biocompatibility [17], reducing corneal damage [18] and enhancing the safety and comfort of wear. As a result, CRT lenses have been widely adopted in the treatment of childhood myopia.

However, there are still certain limitations in the long‐term efficacy and safety evaluations of CRT lenses. Although some short‐term studies have shown that CRT lenses are effective in myopia correction, research on their effects over three years or more, visual quality changes, and long‐term safety is relatively scarce. This study aimed to assess long‐term changes in visual quality, refractive outcomes, and safety during 3 years of CRT lens wear in children with myopia, providing more reliable evidence for clinical treatment.

## 2. Methods

### 2.1. Study Design and Methods

This retrospective study collected data from patients who wore CRT orthokeratology lenses at the ophthalmology outpatient clinic of Beijing Tongren Hospital from February 2021 to November 2024. Detailed longitudinal changes in ocular parameters before and after CRT lens wear, as well as during follow‐up, were recorded, including general ocular, refractive, ocular surface, visual quality, and safety‐related parameters. By analyzing these data, the study comprehensively assessed longitudinal changes in visual quality, refractive outcomes, and safety during CRT lens wear.

This study was conducted in strict accordance with the principles of the Declaration of Helsinki and was approved by the Ethics Committee of Beijing Tongren Hospital (approval number: TREC2023‐KY095.A1). Informed consent was obtained from all participating patients and their parents after they were fully informed about the study.

### 2.2. Participants

#### 2.2.1. Inclusion Criteria

The study selected participants aged 8–18 years with spherical refractive errors between −1.0D and −4.75D. Astigmatism was limited to less than −1.50D. Additionally, both eyes of the participants needed to have a best‐corrected visual acuity (BCVA) of 0.8 or higher. These refractive criteria were selected according to the routine clinical fitting range and design characteristics of the CRT lens system to ensure appropriate lens centration, treatment‐zone formation, and wearing safety. To avoid potential correlations between the two eyes of the same patient that could affect the results, only the right eye of each participant was included in the analysis.

#### 2.2.2. Exclusion Criteria

Children with active ocular inflammation, keratoconus, a history of ocular surgery or trauma, other severe ocular diseases, or use of other myopia control interventions, including atropine eye drops, within 1 month before enrollment were excluded. No additional myopia control interventions were allowed during follow‐up.

### 2.3. Intervention

The orthokeratology lenses used in this study were Paragon CRT 100 lenses (Paragon Vision Sciences, Gilbert, AZ, USA). These lenses feature a unique reverse geometry design that facilitates precise adaptation to the corneal surface, comprising distinct components: the base curve (BC), reverse zone (RZ), and landing zone (LZ). The CRT lenses had an oxygen permeability of 46 × 10^−11^ (cm^2^/s) [mL·O_2_/(mL·hPa)] (ISO units), a 6.00‐mm optical zone (OZ) with a 1.0‐mm return zone width, a reverse zone diameter (RZD) of 6.50–10.00 mm, a center thickness (CT) of 0.15–0.25 mm, and a base curve radius (BCR) of 6.50–10.50 mm.

Subjects were fitted with the lenses according to the manufacturer’s recommendations. Following lens dispensing, participants were instructed to wear their orthokeratology lenses overnight and remove them upon waking, for at least 7 h per night and not to exceed 12 h of total wear per day.

Previous reports showed that corneal thinning can be stabilized by the end of the first week of orthokeratology treatment [19]. Therefore, measurements taken one week after initiating orthokeratology treatment were used as baseline values. Additionally, all patients underwent follow‐up examinations at six‐month intervals after lens wear commenced.

### 2.4. Outcome Measures

At each follow‐up, a comprehensive ocular examination was performed, including (1) **general ocular examination**: slit‐lamp biomicroscopy, intraocular pressure (IOP) assessment, uncorrected visual acuity (UCVA) measurement, BCVA measurement via manifest refraction, and fundus examination; (2) refractive parameters: cycloplegic refraction was performed using four drops of compound tropicamide (0.5% tropicamide + 0.5% phenylephrine; Santen, Japan) administered at 5‐minute intervals; autorefraction (TOPCON KR‐8100, Japan) was conducted 10 min post‐final instillation, with triplicate measurements averaged; axial length (AL) was measured via noncontact partial coherence interferometry (IOL‐Master 500; Carl Zeiss, Germany); AL and refractive measurements were obtained during routine follow‐up while participants continued CRT wear, and no washout period was required, in order to reflect real‐world clinical practice and avoid unnecessary interruption of treatment; (3) ocular surface quality: corneal topography was performed using the Medmont E300 corneal topographer (Medmont International Pty Ltd, Australia) by a single experienced technician; parameters including the corneal surface asymmetry index (SAI), corneal surface regularity index (SRI), tear film surface quality, central tear film surface quality, and CCT were recorded; tear film surface quality and central tear film surface quality were automatically derived from Placido ring–based corneal surface analysis and were used to reflect tear film regularity and central tear film stability; and (4) visual quality parameters: refractive power and corneal analysis were performed using the Optical Path Difference (OPD)‐Scan III (Nidek Co., Ltd., Gamagori, Japan). This device measures full refractive error using dynamic retinoscopy. It simultaneously quantifies total, corneal, and internal aberrations within a 3.0–9.0‐mm pupil area by combining 33‐ring Placido corneal topography with Zernike polynomial wavefront reconstruction. The device’s measurement repeatability and accuracy have been validated [20, 21]. In this study, visual quality was assessed for both the 3‐mm and 5‐mm OZs (the 3‐mm light area represents the pupil size in a bright environment, and the 5‐mm light area represents the pupil size in a dark environment). Parameters such as Strehl ratio (SR), modulation transfer function (MTF), root mean square of total ocular aberrations (total ocular aberration RMS), root mean square of total corneal aberrations (total corneal aberration RMS), root mean square of corneal high‐order aberrations (corneal HOA RMS), root mean square of total intraocular aberrations (total intraocular aberration RMS), root mean square of corneal spherical aberration (corneal SA RMS), root mean square of corneal coma (corneal coma RMS), root mean square of corneal trefoil (corneal trefoil RMS), and root mean square of corneal tilt (corneal tilt RMS) were recorded.

During each follow‐up, the doctor also inquired in detail about the patient’s lens‐wearing experience, including wear time, comfort, and any ocular discomfort. An ocular examination was performed to check for any abnormal corneal changes, such as epithelial damage or corneal edema. Any issues that arose were promptly addressed and documented.

### 2.5. Statistical Analysis

Statistical analysis was performed using SPSS Version 26.0 (SPSS Inc., USA). Quantitative data were expressed as mean ± standard deviation (M ± SD), and ordinal data were described using the median (interquartile range). For the outcome measures, the Shapiro–Wilk test was applied to assess the normality of the distribution of the measured variables. Repeated measures analysis of variance (ANOVA) was used to analyze the data across the three follow‐up visits, and post hoc comparisons were conducted using the Bonferroni test to reduce the risk of Type I error, with statistical significance set at *p* < 0.0167. A paired‐samples *t* test was used to compare the indicators of the 3‐mm OZ and the 5‐mm OZ, with statistical significance set at *p* < 0.05. All statistical tests were two‐sided.

## 3. Results

### 3.1. Participant Characteristics

A total of 130 subjects, corresponding to 130 eyes, were included in the statistical analysis. To eliminate the between‐eye correlation, the right eyes of the subjects were included in this study. The mean age was 12.35 ± 4.27 years (range: 8–18 years), with 67 females (51.54%) and 63 males (48.46%). Spherical power (DS) ranged from −1.00 D to −4.75 D (mean, −1.36 ± 1.16 D), and cylindrical power (DC) was < 1.50 D (mean, −0.70 ± 0.83 D). BCVA was 16/20 or better (Table 1).

**TABLE 1:** Baseline characteristics of the patients.

	***n*/*x***	**%/s**

Age (year)	12.35	4.27
Gender		
Female	67	51.54
Male	63	48.46
DS (D)	−1.36	1.17
DC (D)	−0.70	0.83
AL (mm)	24.62	0.83
BCVA	0.98	0.09

*Note:* DS: diopter of spherical power; DC: diopter of cylindrical power.

Abbreviations: AL, axial length; BCVA, best‐corrected visual acuity.

### 3.2. Efficacy Analysis

During the 3‐year follow‐up period, both the myopic refractive error and AL showed a slight increase. At baseline, the relevant parameters of the patients are shown in Table 1. After one and a half years of wear, the mean DS increased to −1.42 ± 1.14 D, the mean DC increased to −0.91 ± 0.79D, and the AL increased to 24.85 ± 0.83 mm. By the three‐year follow‐up, the mean DS had further increased to −1.69 ± 1.24D, the mean DC increased to −1.18 ± 0.99D, and the AL increased to 25.08 ± 0.85 mm. After three years of wearing CRT orthokeratology lenses, the patients’ BCVA remained almost unchanged (Figure 1).

FIGURE 1Myopia progression after three years of wearing CRT orthokeratology lenses: (a) spherical equivalent, (b) cylindrical power, (c) axial length, and (d) best‐corrected visual acuity.

**Figure:**
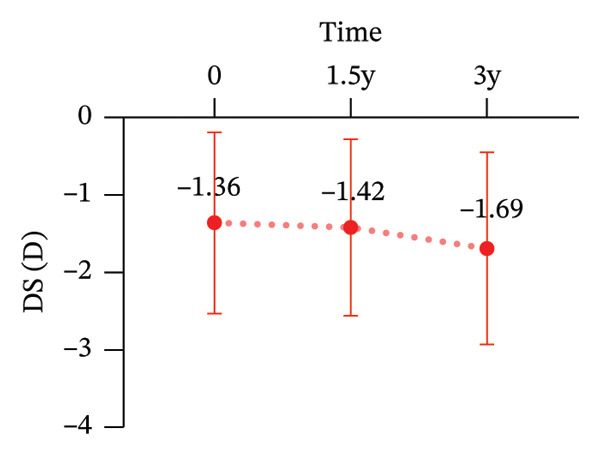
(a)

**Figure:**
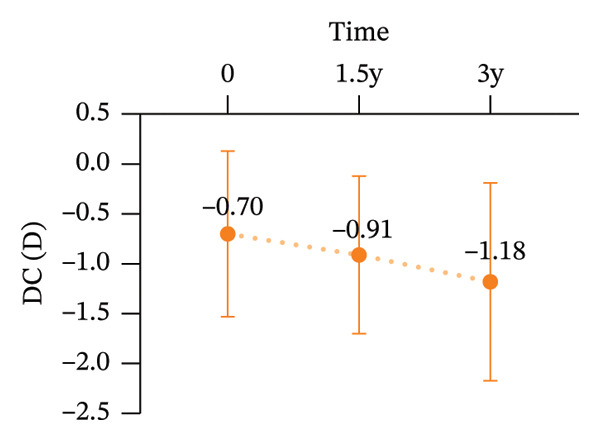
(b)

**Figure:**
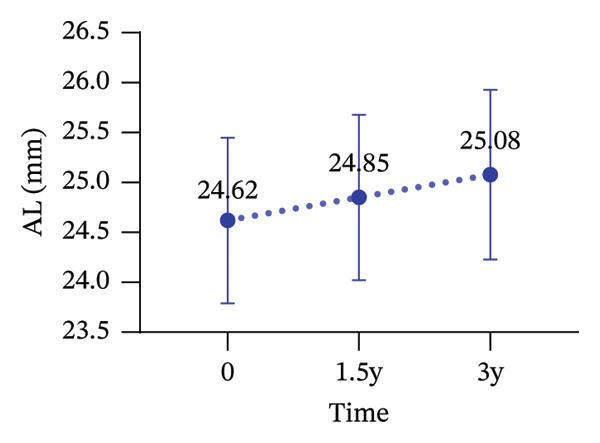
(c)

**Figure:**
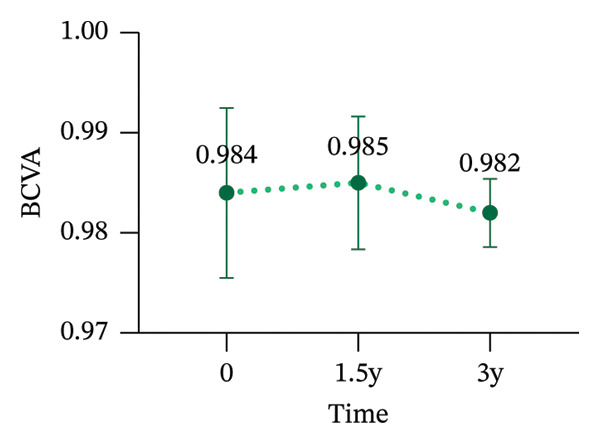
(d)

### 3.3. Visual Quality Indicators

Changes in ocular and corneal visual quality over the 3‐year follow‐up are presented in Figure 2, with detailed quantitative results shown in Table 2.

FIGURE 2Changes in ocular and corneal visual quality after 3 years of CRT orthokeratology lens wear: (a) 3‐mm optical zone; (b) 5‐mm optical zone.

**Figure:**
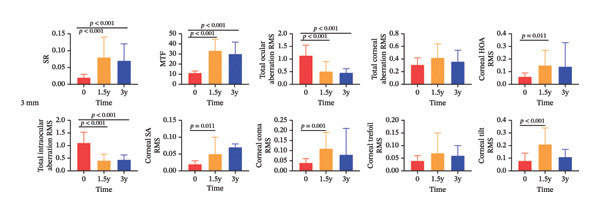
(a)

**Figure:**
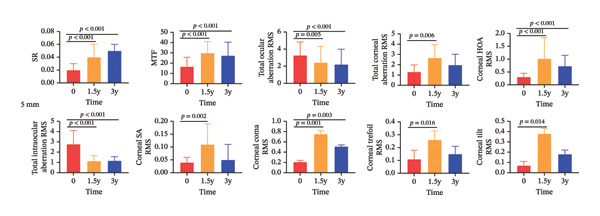
(b)

**TABLE 2:** Visual quality at three follow‐up visits.

	**Visit**	**3 mm**		**5 mm**		**3 vs. 5 mm**	
		**x ± s**	**p** **(vs. baseline)**	**x ± s**	**p** **(vs. baseline)**	**t**	**p**
SR	Baseline	0.02 ± 0.01		0.02 ± 0.01		−0.902	0.375
	1.5 y	0.08 ± 0.06	**< 0.001**	0.04 ± 0.02	**< 0.001**	6.767	**< 0.001**
	3 y	0.07 ± 0.05	**< 0.001**	0.05 ± 0.01	**< 0.001**	5.973	**< 0.001**

MTF	Baseline	11.37 ± 1.75		16.68 ± 9.08		−2.964	**0.006**
	1.5 y	33.33 ± 11.30	**< 0.001**	29.89 ± 11.10	**< 0.001**	2.114	**0.044**
	3 y	30.06 ± 11.73	**< 0.001**	27.34 ± 13.08	**< 0.001**	1.608	0.119

Total ocular aberration RMS	Baseline	1.14 ± 0.41		3.28 ± 1.55		−8.978	**< 0.001**
	1.5 y	0.51 ± 0.39	**< 0.001**	2.43 ± 1.88	0.005	−6.442	**< 0.001**
	3 y	0.46 ± 0.16	**< 0.001**	2.21 ± 1.77	**< 0.001**	−8.603	**< 0.001**

Total corneal aberration RMS	Baseline	0.31 ± 0.11		1.31 ± 0.67		−4.270	**< 0.001**
	1.5 y	0.42 ± 0.22	0.040	2.67 ± 1.27	**0.006**	−5.821	**< 0.001**
	3 y	0.36 ± 0.18	0.323	1.98 ± 1.04	0.027	−8.765	**< 0.001**

Corneal HOA RMS	Baseline	0.06 ± 0.03		0.31 ± 0.14		−3.371	**0.002**
	1.5 y	0.15 ± 0.12	**0.011**	1.02 ± 0.81	**< 0.001**	−6.692	**< 0.001**
	3 y	0.14 ± 0.19	0.203	0.73 ± 0.42	**< 0.001**	−7.439	**< 0.001**

Corneal SA RMS	Baseline	0.02 ± 0.01		0.04 ± 0.02		−3.127	**0.004**
	1.5 y	0.05 ± 0.05	**0.011**	0.11 ± 0.08	**0.002**	−3.297	**0.003**
	3 y	0.07 ± 0.01	0.062	0.05 ± 0.06	0.780	−1.157	0.257

Corneal coma RMS	Baseline	0.04 ± 0.02		0.21 ± 0.03		−2.686	**0.012**
	1.5 y	0.11 ± 0.08	**0.001**	0.75 ± 0.07	**0.001**	−5.326	**< 0.001**
	3 y	0.08 ± 0.13	0.288	0.51 ± 0.03	**0.003**	−6.009	**< 0.001**

Corneal trefoil RMS	Baseline	0.04 ± 0.02		0.11 ± 0.07		−5.687	**< 0.001**
	1.5 y	0.07 ± 0.08	0.202	0.26 ± 0.07	**0.016**	−5.878	**< 0.001**
	3 y	0.06 ± 0.04	0.621	0.15 ± 0.06	0.588	−7.074	**< 0.001**

Corneal tilt RMS	Baseline	0.08 ± 0.06		0.07 ± 0.04		−2.661	**0.013**
	1.5 y	0.21 ± 0.13	**< 0.001**	0.38 ± 0.05	**0.014**	−4.121	**< 0.001**
	3 y	0.11 ± 0.06	0.188	0.18 ± 0.04	0.122	−5.858	**< 0.001**

Total intraocular aberration RMS	Baseline	1.11 ± 0.42		2.78 ± 1.32		−8.482	**< 0.001**
	1.5 y	0.41 ± 0.25	**< 0.001**	1.14 ± 0.52	**< 0.001**	−10.140	**< 0.001**
	3 y	0.44 ± 0.19	**< 0.001**	1.17 ± 0.39	**< 0.001**	−11.382	**< 0.001**

*Note:* 1.5 y and 3 y vs. baseline: Post hoc Bonferroni test was performed, and a *p* value < 0.0167 was considered statistically significant. 3 vs. 5 mm: *p* < 0.05 was considered statistically significant. Total ocular aberration RMS: root mean square of total ocular aberrations; total corneal aberration RMS: root mean square of total corneal aberrations; corneal HOA RMS: root mean square of corneal high‐order aberrations; total intraocular aberration RMS: root mean square of total intraocular aberrations; corneal SA RMS: root mean square of corneal spherical aberration; corneal coma RMS: root mean square of corneal coma; corneal trefoil RMS: root mean square of corneal trefoil; corneal tilt RMS: root mean square of corneal tilt. Bold values indicate statistical significance. For comparisons between 1.5 years or 3 years and baseline, post hoc Bonferroni tests were performed, with a significance threshold of *p* < 0.0167. For comparisons between 3 mm and 5 mm pupil diameters, *p* < 0.05 was considered statistically significant.

Abbreviations: MTF, modulation transfer function; SR, Strehl ratio.

In terms of total ocular visual quality, SR and MTF within the 3‐mm and 5‐mm OZs showed a significant upward trend at both 1.5 years and 3 years of treatment (both *p* < 0.001), while total ocular aberration RMS significantly decreased (both *p* < 0.01). Compared to the 5‐mm OZ, the 3‐mm OZ exhibited higher SR and MTF (both *p* < 0.001) and lower total ocular aberration RMS (*p* < 0.001) at both 1.5 and 3 years, except for MTF at 3 years.

Regarding corneal visual quality, total corneal aberration RMS showed no significant difference in the 3‐mm OZ but significantly increased in the 5‐mm OZ (both *p* < 0.001), with values significantly higher in the 5‐mm zone than in the 3‐mm zone (*p* < 0.001). At 1.5 years, corneal HOA RMS, corneal SA RMS, corneal coma RMS, and corneal tilt RMS significantly increased in the 3‐mm and 5‐mm OZs (*p* = 0.011; *p* = 0.011; *p* = 0.001; *p* < 0.001; *p* < 0.001; *p* = 0.002; *p* = 0.001; *p* = 0.014). At 3 years, all corneal higher‐order aberrations in the 3‐mm OZ increased compared to baseline, but the differences were not significant. However, in the 5‐mm OZ, corneal HOA RMS and corneal coma RMS were significantly higher than baseline (*p* < 0.001; *p* = 0.003), and overall, corneal higher‐order aberrations were significantly greater in the 5‐mm OZ than in the 3‐mm zone.

Total intraocular aberration RMS significantly decreased in both the 3‐mm and 5‐mm OZs at 1.5 and 3 years (both *p* < 0.001), with values significantly lower in the 3‐mm zone than in the 5‐mm zone (*p* < 0.001). After three years of wear, the corneal SAI increased (*p* < 0.001), while the tear film surface quality and central tear film surface quality remained unchanged (*p* > 0.05).

### 3.4. Safety‐Related Indicators

During the 3‐year follow‐up, no cases of microbial keratitis, corneal edema, or other severe corneal complications were observed. Mild corneal staining was noted in 4 patients (3.1%), and mild conjunctival hyperemia occurred in 2 patients (1.5%). These mild adverse events resolved after temporary discontinuation of lens wear for approximately 1 week. In addition, corneal endothelial cell density remained stable throughout follow‐up, with no significant change over time (all *p* > 0.05).

## 4. Discussion

Myopia is a common ocular condition globally, with an increasing prevalence among children [22]. It not only affects daily activities and academic performance but may also have long‐term adverse effects on visual function and overall quality of life [23, 24]. As a nonsurgical method of myopia correction, orthokeratology lenses, due to their unique corrective mechanism and significant myopia control effects, have been widely applied in clinical practice [25, 26]. Among the available designs, CRT lenses, with their reverse‐geometry design and high oxygen‐permeable materials, have been widely adopted in pediatric myopia management [27]. By temporarily reshaping the cornea, CRT lenses can reduce myopic refractive error and improve UCVA and have also been reported to slow axial elongation in children [8, 28].

Over 3 years of follow‐up in children wearing CRT lenses, this study yielded several notable findings regarding visual quality, refractive outcomes, and safety. (1) After three years of CRT lens wear, patients showed improvements in total ocular visual quality in both the 3‐mm and 5‐mm OZs: the SR and MTF increased, and total ocular aberration RMS decreased, with better performance in the 3‐mm OZ. (2) However, regarding corneal aberrations, total corneal aberration RMS, corneal HOA RMS, corneal SA RMS, corneal coma RMS, and corneal tilt RMS increased to varying extents in both OZs, with more pronounced changes in the 5‐mm OZ. (3) Total intraocular aberration RMS significantly decreased in both the 3‐mm and 5‐mm OZs, with better performance in the 3‐mm zone. (4) During the three‐year follow‐up, the DS increased by an average of −0.33D, the DC increased by an average of −0.48D, and the AL increased by an average of 0.46 mm. These changes were broadly consistent with previously reported orthokeratology outcomes [29, 30].

Although no direct comparison was possible in the present study, the axial elongation observed in this cohort appeared numerically lower than values reported for untreated myopic children in previous studies [28, 31]. By reshaping the central cornea, orthokeratology lenses create a peripheral defocus ring, which induces peripheral myopic defocus. This defocus can inhibit axial elongation by modulating retinal signaling pathways [32]. In addition, the geometric fitting characteristics of CRT lenses allow precise corneal alignment through adjustment of the RZD, which may enhance central corneal flattening and contribute to myopia control [33].

Our study demonstrated that total corneal aberrations (total corneal aberration RMS) and HOA in the 5‐mm OZ significantly increased after CRT orthokeratology lens wear, with values consistently higher than those in the 3‐mm OZ. This phenomenon is closely associated with the “central flattening with midperipheral steepening” design of orthokeratology lenses [34]. As widely recognized, myopic orthokeratology applies positive pressure centrally and negative pressure peripherally under the steeper secondary reverse curve, flattening the central corneal treatment zone to reduce corneal power for refractive correction [35]. The midperipheral compression induces centripetal migration of peripheral corneal epithelial cells, forming a steepened region outside the treatment zone [36–38], which leads to asymmetric corneal curvature changes and consequently elevates asymmetric aberrations such as coma and trefoil. Furthermore, the broader coverage of the 5‐mm OZ renders it more susceptible to peripheral corneal asymmetric remodeling [39], whereas the 3‐mm OZ primarily reflects the central corneal optical properties, which stabilize over long‐term wear.

Despite increased corneal aberrations, total intraocular aberrations (total intraocular aberration RMS) significantly decreased in both 3‐mm and 5‐mm OZs. The reduction in intraocular aberrations may originate from dynamic adjustments of the crystalline lens [40], partially neutralizing orthokeratology‐induced higher‐order aberrations such as SA and coma [34]. Additionally, CRT lenses improve retinal image quality through central OZ remodeling while mitigating the visual impact of higher‐order aberrations via peripheral defocus rings [31, 41].

The significant reduction in total ocular aberrations and the elevation of SR and MTF reflect the compensatory optimization of the ocular optical system by intraocular mechanisms. Adaptive adjustments in intraocular aberrations (e.g., crystalline lens modifications) effectively counteract corneal aberration increases [40], thereby reducing overall wavefront aberrations. Moreover, long‐term visual feedback may enhance neural adaptation to residual aberrations [42], contributing to improved perceptual image quality. This multilevel compensatory mechanism enhances MTF and SR in both OZs, particularly under daytime photopic conditions (3‐mm pupil), where patients achieve superior high‐contrast acuity due to minimized peripheral aberration interference [42].

During follow‐up, only a small number of mild, reversible ocular surface events were observed, mainly corneal staining and conjunctival hyperemia, and no microbial keratitis or other severe corneal complications occurred. Additionally, there was no significant decline in tear film surface quality or central tear film surface quality. Previous studies have similarly confirmed the good safety profile of CRT lenses [17, 43]. This may be attributed to the material used in CRT lenses, which features a smaller wettability angle (enhancing comfort) and high oxygen transmissibility, thereby reducing the risk of corneal hypoxia and epithelial damage [44].

This study provided a relatively comprehensive assessment of long‐term changes in visual quality, safety, and refractive outcomes during CRT lens wear. Nevertheless, several limitations should be acknowledged. First, as a single‐center, single‐arm study without a control group, it did not allow direct comparison of the efficacy of CRT lenses with other myopia control strategies. Second, visual quality was assessed mainly using objective parameters, whereas subjective visual experience was not evaluated in sufficient depth. Patient‐reported outcomes, including visual comfort and visual fatigue, are also important for determining the practical clinical value of CRT lenses. Third, because the study population was restricted to children within the routine fitting range of the CRT system, the generalizability of the findings to patients with higher myopia or greater astigmatism may be limited.

## 5. Conclusion

This study indicates that long‐term CRT lens wear may improve overall and intraocular visual quality, despite increased corneal higher‐order aberrations, with better optical performance observed in smaller OZs. In addition, CRT lenses demonstrated a favorable safety profile in this cohort, and the 3‐year axial elongation and refractive changes were consistent with previously reported orthokeratology outcomes.

## Author Contributions

Shang Li and Yihan Guo contributed equally to this work and share first authorship. Conceptualization: Ying Jie. Methodology: Shang Li, Lei Tian, and Ying Jie. Formal analysis: Shang Li and Yihan Guo. Data curation: Shang Li, Yihan Guo, Qianru Wu, and Ao Li. Writing–original draft: Shang Li and Yihan Guo. Writing–review and editing: Shang Li, Lei Tian, and Ying Jie. Supervision: Ying Jie.

## Funding

This study was supported by “Yicheng Outstanding Talents” in Beijing Economic‐Technological Development Area, Beijing Hospitals Authority’s Ascent Plan (grant No. DFL20240202), and MIIT High‐Quality Development Special Program for Manufacturing, Key Task 35‐2024: Human Ocular Wavefront Aberration Measurement System Project.

## Disclosure

All authors have read and approved the final manuscript. All authors contributed to the article and approved the submitted version.

## Ethics Statement

The study was conducted in accordance with the aims of the Declaration of Helsinki. The study involving human participants was reviewed and approved by the Ethics Committee of Beijing Tongren Hospital (board approval number: TREC2023‐KY095.A1). The patients/participants provided their written informed consent to participate in this study.

## Conflicts of Interest

The authors declare no conflicts of interest.

## Data Availability

The data that support the findings of this study are available from the corresponding author upon reasonable request.
